# Prediction of the Trimer Protein Interface Residue Pair by CNN-GRU Model Based on Multi-Feature Map

**DOI:** 10.3390/nano15030188

**Published:** 2025-01-24

**Authors:** Yanfen Lyu, Ting Xiong, Shuaibo Shi, Dong Wang, Xueqing Yang, Qihuan Liu, Zhengtan Li, Zhixin Li, Chunxia Wang, Ruiai Chen

**Affiliations:** 1 College of Veterinary Medicine, South China Agricultural University, Guangzhou 510642, China; lyuyf@hebeu.edu.cn (Y.L.); bearvet@163.com (T.X.); 2 School of Mathematics and Physics, Hebei University of Engineering, Handan 056038, China; shi258329@163.com (S.S.); yjjmon@163.com (X.Y.); lqh092296@163.com (Q.L.); lzt112496@163.com (Z.L.); lizhixin@hebeu.edu.cn (Z.L.); 3 Key Laboratory of Manufacture Technology of Veterinary Bioproducts, Ministry of Agriculture and Rural Affairs, Zhaoqing Dahuanong Biology Medicine Co., Ltd., Zhaoqing 526238, China; 4 Zhaoqing Branch of Guangdong Laboratory of Lingnan Modern Agricultural Science and Technology, Zhaoqing 526238, China; 5 School of Mechanical and Equipment Engineering, Hebei University of Engineering, Handan 056038, China; wangdong45@hebeu.edu.cn; 6 College of Landscape and Ecological Engineering, Hebei University of Engineering, Handan 056038, China

**Keywords:** trimer protein, CNN-GRU, multi-feature map

## Abstract

Most life activities of organisms are realized through protein–protein interactions, and these interactions are mainly achieved through residue–residue contact between monomer proteins. Consequently, studying residue–residue contact at the protein interaction interface can contribute to a deeper understanding of the protein–protein interaction mechanism. In this paper, we focus on the research of the trimer protein interface residue pair. Firstly, we utilize the amino acid k-interval product factor descriptor (AAIPF(k)) to integrate the positional information and physicochemical properties of amino acids, combined with the electric properties and geometric shape features of residues, to construct an 8 × 16 multi-feature map. This multi-feature map represents a sample composed of two residues on a trimer protein. Secondly, we construct a CNN-GRU deep learning framework to predict the trimer protein interface residue pair. The results show that when each dimer protein provides 10 prediction results and two protein–protein interaction interfaces of a trimer protein needed to be accurately predicted, the accuracy of our proposed method is 60%. When each dimer protein provides 10 prediction results and one protein–protein interaction interface of a trimer protein needs to be accurately predicted, the accuracy of our proposed method is 93%. Our results can provide experimental researchers with a limited yet precise dataset containing correct trimer protein interface residue pairs, which is of great significance in guiding the experimental resolution of the trimer protein three-dimensional structure. Furthermore, compared to other computational methods, our proposed approach exhibits superior performance in predicting residue–residue contact at the trimer protein interface.

## 1. Introduction

Proteomics is a popular but complex area in bioinformatics research, with the study of protein–protein interactions constituting a vital component of proteomics research. Through the study of protein–protein interactions, we can gain a deeper understanding of their interaction mechanisms at the atomic level, providing guidance and assistance for the experimental resolution of the three-dimensional structures of ultra-large multi-body protein complexes [1,2,3]. The binding sites involved in these interactions may also be functional sites, which can be used as potential drug targets and help to design functional proteins. A variety of experimental techniques are available for the study of protein–protein interactions, such as the yeast two-hybrid method [4], cryogenic electron microscopy [5], X-ray crystallography [6], and nuclear magnetic resonance [7]. Experimental methods possess non-negligible defects, such as being time-consuming, which greatly reduce the feasibility of these methods in studying all protein–protein interactions. Therefore, we need to develop computational methods to study protein–protein interactions.

At present, numerous computational methods have been proposed for the study of protein–protein interactions and protein binding sites. For example, Baranwal et al. used a graph attention network method to study protein–protein interactions [8]. Fu et al. utilized an attention mechanism and graph convolutional networks to study protein binding sites [9]. Additionally, numerous studies, such as [10,11,12,13], also focus on the research of protein–protein interactions or protein binding sites. With the AlphaFold2 method proposed by the DeepMind team, the prediction of the monomer protein structure has been essentially solved [14]. However, most life activities are achieved through multi-body protein interactions. For example, the protein capsids of many viral shells are assembled often from the dimers-of-trimers of capsid–protein units (e.g., HCoV-OC43 and infectious bronchitis virus) [15,16,17,18]. Exploring and studying these multi-body protein interactions will provide us with new perspectives and clues regarding the mysteries of life, as well as new ideas and methods for the prevention and treatment of diseases. The DeepMind team, having further optimized its algorithm based on the AlphaFold2 method, has proposed the AlphaFold3 method, which significantly enhances the prediction accuracy compared to the AlphaFold2 method. However, this method still faces challenges in modeling multi-target proteins [19]. Minkyung Baek et al. conducted an in-depth analysis of the features in the AlphaFold2 method that play an important role in protein structure research. Based on these important features, they improved the RoseTTAFold method, proposing the RoseTTAFold2 method [20]. This method can be applied to the study of homodimers and heterodimer structures, but, when dealing with large protein complex structures, due to the existence of cropping issues, the prediction results of this method are not satisfactory. Pierce B et al. proposed the M-Dock docking method based on the Fast Fourier Transform (FFT) for the prediction of multi-body protein structures and provided the corresponding server. However, the M-Dock method is only applicable in predicting multi-body proteins with symmetrical structures [21]. Zhao et al. attempted to use LSTM networks to study trimer protein interactions, but their prediction results were not high [22]. Lyu et al. designed a two-layer SVM model to study residue–residue contact at the trimer protein interface [23]. Subsequently, Lyu et al. utilized convolutional neural networks to explore the interaction mechanisms among the individual chains within the tetramer protein. Building upon this foundation, they further used the support vector machine method to predict the interface residue pairs of two chains [24]. In this paper, we develop a method for the study of interface residue pairs in trimer proteins, further improving the prediction accuracy.

The protein sequence contains implicit information about the protein structure and function, which may be hidden in the properties, structures, and positions of the twenty amino acids that make up the protein sequence. Exploiting this hidden information is crucial in predicting protein binding sites. Previous research has shown that the physicochemical properties of amino acids (e.g., polarity), as well as the geometric shape characteristics of residues (e.g., exterior void area (EVA)), coupled with the arrangement positions of amino acids within the protein sequence, play crucial roles in studying the structure and function of proteins [25,26]. We utilize the above three categories of properties of amino acids to study the interface residue pairs of the trimer protein.

In this paper, we study the interface residue pairs of the trimer protein in three steps. The first step is feature extraction, where we utilize three important properties of amino acids (physicochemical, electric, and geometric shape properties), and take into account the influence of other amino acids based on distance, to extract 64 features representing a residue. The second step is to construct multi-feature maps. Based on the features extracted previously, we build an 8 × 16 multi-feature map representing a sample composed of two residues on a trimer protein. The third step is to generate a CNN-GRU deep learning model. Within the CNN component, we also use Bayesian optimization to adjust the model’s hyperparameters.

## 2. Materials and Methods

### 2.1. Dataset Preparation

We downloaded trimer protein data from the Protein Data Bank and screened the data based on the following three conditions: (1) each protein complex contained three chains, with each chain having between 20 and 500 amino acids; (2) the three-dimensional structure of the protein complex must be precisely determined by X-ray crystallography; (3) it was required that there was an interaction between any two chains within each trimer protein. If the calculation results of the Qcontacts software (version 0.112881) showed that there were two residues with a contact area greater than zero on the two chains in the protein complex, it was considered that there was an interaction between the two chains. These two residues are also referred to as interface residue pairs. After the screening, we obtained 78 trimer proteins.

In this paper, a sample refers to a residue pair formed by any two residues on two chains of a trimer protein. Positive and negative samples refer to the interface residue pairs and non-interface residue pairs of the protein trimer, respectively. We generated a total of 10,342,333 samples using 78 trimer proteins, with 18,818 positive samples and 10,323,515 negative samples. The positive samples accounted for approximately 0.182% of the total samples. We randomly divided the data into three parts (a training set, a validation set, and a test set) at a ratio of 2:1:1. The PDB IDs of the trimer proteins and detailed information about the samples in each dataset are listed in Tables S1–S3.

### 2.2. Methods

#### 2.2.1. Feature Extraction

Amino acids are the basic components of proteins, and all types of proteins in organisms are composed of 20 amino acids, which are called standard amino acids. We use set Ω={A,C,D,E,F,G,H,I,K,L,M,N,P,Q,R,S,T,V,W,Y} to represent the standard amino acid set, where the elements in set Ω represent the standard amino acids. We utilize Formula (1) to denote the primary sequence of protein P, where L represents the count of amino acids within the protein, and t represents the positions of the amino acids in the protein’s primary sequence.

(1) $$P=A_{1}A_{2}{\cdots A}_{t}\cdots A_{L}\left(A_{t}\in \Omega ,t=1,2,\cdots L\right)$$

Amino acids exhibit different physicochemical properties due to their different side chains. According to references [27,28,29,30], it has been found that the amino acids’ hydrophobicity, polarizability, polarity, secondary structure, and codon diversity play important roles in protein complex structure studies. The hydrophobicity of amino acids was proposed by Tanford in 1962, by Kyte in 1982, and by Eisenberg in 1984, respectively [31,32,33].

These three types of hydrophobicity values have demonstrated exceptional performance in exploring proteins’ functional sites and protein binding sites. Therefore, we utilize the five physicochemical properties of amino acids mentioned above (including the three versions of hydrophobicity) to research the interface residue pairs of the trimer protein. We use $\rho^{1}$, $\rho^{2}$, $\rho^{3}$, $\rho^{4}$, $\rho^{5}{,\rho }^{6}$ and $\rho^{7}$ to represent the different physicochemical properties of amino acids, as shown in Table S4. We label the three versions of hydrophobicity as Hydrophobicity 1, Hydrophobicity 2, and Hydrophobicity 3, respectively.

The protein P can be transformed into seven numerical sequences based on the aforementioned properties of amino acids, as illustrated in Formula (2). $P^{1}$ represents the hydrophobicity number sequence 1 of protein P, $P^{2}$ represents the polarizability number sequence of protein P, $P^{3}$ represents the polarity number sequence of protein P, $P^{4}$ represents the secondary structure number sequence of protein P, $P^{5}$ represents the codon diversity number sequence of protein P, $P^{6}$ represents the hydrophobicity number sequence 2 of protein P, and $P^{7}$ represents the hydrophobicity number sequence 3 of protein P.

(2) $$P^{i}=\rho_{1}^{i}\rho_{2}^{i}\cdots \rho_{L}^{i}(i=1,2,3,4,5,6,7)$$

In a protein complex, each amino acid is influenced to varying degrees by other amino acids, and the strength of this influence is closely related to the distance between them in the sequence. In our previous research, in order to quantify and characterize the distance-based influence among amino acids, we defined the concept of the amino acid k-interval product factor (AAIPF(k)) to describe the influences of other amino acids on a given amino acid [23]. In this paper, we also employ this to extract features. Its calculation formula is given in Formulas (3)–(5). In Formulas (3)–(5), t represents the position of a given amino acid in the protein sequence, and k represents the distance between other amino acids and the given amino acid in the protein sequence.

(3) $$AAIPF\left(k\right)=\left\{\begin{array}{c} AAFIPF\left(k\right)\\ AABIPF\left(k\right) \end{array}\right.$$

(4) $$AAFIPF\left(k\right)=\frac{\rho_{t}^{i}{\times \rho }_{t-k}^{i}}{k}\left(i=1,2,3,4,5,6,7\right)$$

(5) $$AABIPF\left(k\right)=\frac{\rho_{t}^{i}\times \rho_{t+k}^{i}}{k}\left(i=1,2,3,4,5,6,7\right)$$

Considering the redundancy of features, we combine previous research experience [23,34,35] and set four conditions when extracting features. First, the primary sequence of the protein is regarded as a circular sequence. Second, for any given amino acid, we only focus on the effect of the 5 positions before and after it. Third, when calculating the amino acid k-interval product factor (AAIPF(k)), we use five numerical sequences with different properties, among which the hydrophobicity is selected as one of the three versions of the numerical sequence. Fourth, we adopt the values of the 5 physicochemical properties of amino acids (including the 3 versions of hydrophobicity) as the 7 basic sequence features to characterize an amino acid. Thus, we extract a total of 57 features and combine them into the vector $V_{t1}$, as shown in Formula (6).

(6) $$V_{t1}=\left({AAIPF}_{t}^{i}\left(1\right),{AAIPF}_{t}^{i}{\left(2\right),AAIPF}_{t}^{i}\left(3\right),{AAIPF}_{t}^{i}\left(4\right),{AAIPF}_{t}^{i}\left(5\right),\rho_{t}^{j}\right)\;i\in \left[1,5\right],j\in \left[1,7\right]$$

The binding rate and the stable protein complex structure formed during protein–protein interactions are both influenced by electrostatic interactions. Therefore, we regard the electric properties (${pk}_{1}$ and ${pk}_{2}$) as a group of features of residues. We can use the software propka3.1 to calculate these two features (${pk}_{1}$ and ${pk}_{2}$) of residues [23,36]. We combine ${pk}_{1}$ and ${pk}_{2}$ into the vector $V_{t2}$, as shown in Formula (7).

(7) $$V_{t2}=\left({pk}_{t1},{pk}_{t2}\right)$$

The geometric shape properties of residues also help to identify the binding sites of protein complexes. For instance, in reference [37], the researchers used the accessible surface area (ASA) and relative accessible surface area (RASA) to predict monomer protein binding sites, yielding promising results. Reference [38] used three geometric shape features, the external contact area (EA), internal contact area (IA), and external void area (EVA), to identify interface residue pairs in protein complexes and achieved promising prediction results. Therefore, we use the above five geometric shape features to describe each residue.

ASA stands for the solvent-accessible surface area of a molecule. RASA is utilized to characterize the exposed or buried state of residues, and its calculation is shown in Formula (8). EA represents the contact area between a residue and its surrounding residues. IA stands for the contact area between different atoms within a residue. EVA represents the non-contact area, explicitly referring to the portion of a residue that maintains no contact with either solvents or other residues. In this paper, we use the Naccess V2.1.1 and Qcontacts software, combined with self-coded programs, to calculate the above five geometric shape features [39,40]. These five geometric shape features are then combined to form a vector $V_{t3}$, as shown in Formula (9). The usage of the Qcontacts software is as follows: (1) download and install the Qcontacts software package; (2) run the software; (3) input the protein PDB ID, the chain 1 name, and the chain 2 name; (4) the software will automatically calculate and output the contact area between the atoms within a residue and the contact area between atoms from two residues on two chains.

(8) $$RASA=\frac{{ASA}_{bound}}{{ASA}_{unbound}}$$

(9) $$V_{t3}=\left({ASA}_{t},{RASA}_{t},{EA}_{t},{IA}_{t},{EVA}_{t}\right)$$

#### 2.2.2. Constructing Multi-Feature Maps

**Figure 1:**
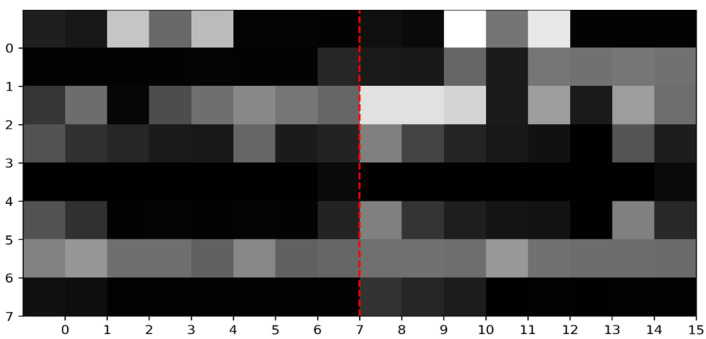
Multi-feature map of a sample.

According to Section 2.2.1 on feature extraction, we obtain a total of 64 features to characterize a residue. Then, these 64 features are transformed into an 8 × 8 matrix to represent a residue, as shown in Formula (10). By concatenating the feature matrices of two residues, we construct an 8 × 16 feature matrix that represents a sample composed of these two residues. Taking into account the varying scales of the feature values, we employ the min-max normalization method to normalize each sample’s 8 × 16 feature matrix, ensuring that all feature values are scaled to fall within the range of 0 to 1, as shown in Formula (11). We consider the normalized feature matrix as a grayscale image, where higher values correspond to brighter pixels and lower values correspond to darker pixels. We define this grayscale image as a multi-feature map; see Figure 1.

(10) $$\left[\begin{array}{ccc} {\mathrm{AAFIPF}}_{t}^{1}\left(1\right) & \text{⋯} & {\mathrm{AABIPF}}_{t}^{1}\left(3\right)\\ \text{⋮} & \text{⋱} & \text{⋮}\\ \rho_{t}^{7} & \text{⋯} & {\mathrm{EVA}}_{t} \end{array}\right]$$

(11) $$x^{*}=\frac{x-x_{min}}{x_{max}-x_{min}}$$

#### 2.2.3. Generating CNN-GRU Model

**Figure 2:**
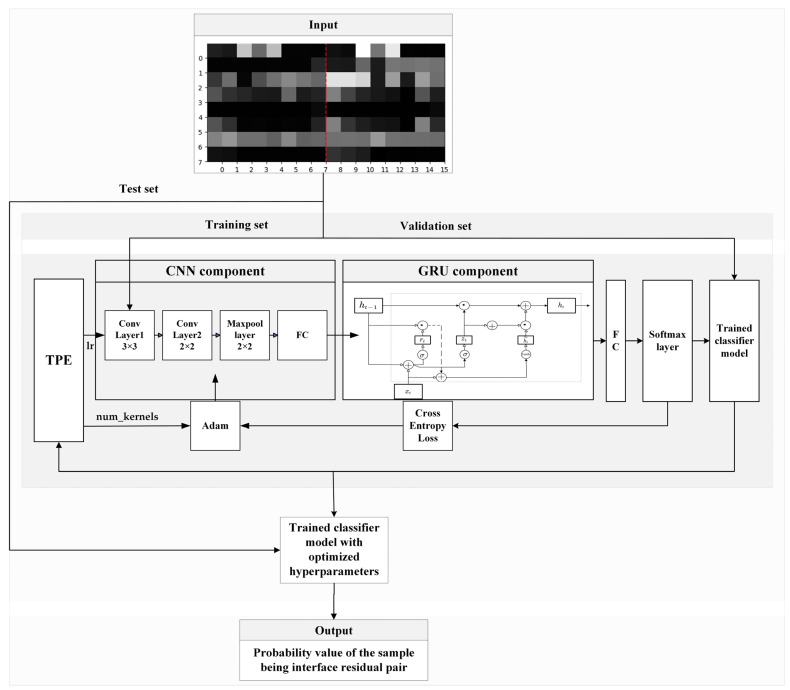
CNN-GRU model network architecture.

As a type of feedforward neural network, a CNN processes and recognizes data by progressively passing information through layers [41]. The convolutional layer is the core component of the CNN, which uses convolutional kernels to scan the input data to extract local features. The primary function of the pooling layer in the CNN is to reduce the spatial dimensions of the data. The fully connected layer in the CNN is responsible for receiving features extracted from previous layers and synthesizing them to produce the final output. A GRU is a recurrent neural network based on a gating mechanism, which balances the memorization and forgetting of historical information by introducing update and reset gates, thus solving the problems of gradient vanishing and exploding in traditional RNNs when dealing with long-term dependencies [42]. We designed a CNN-GRU model based on a CNN and GRU to predict the interface residue pairs of the trimer protein, with its network architecture illustrated in Figure 2.

**Figure 3:**
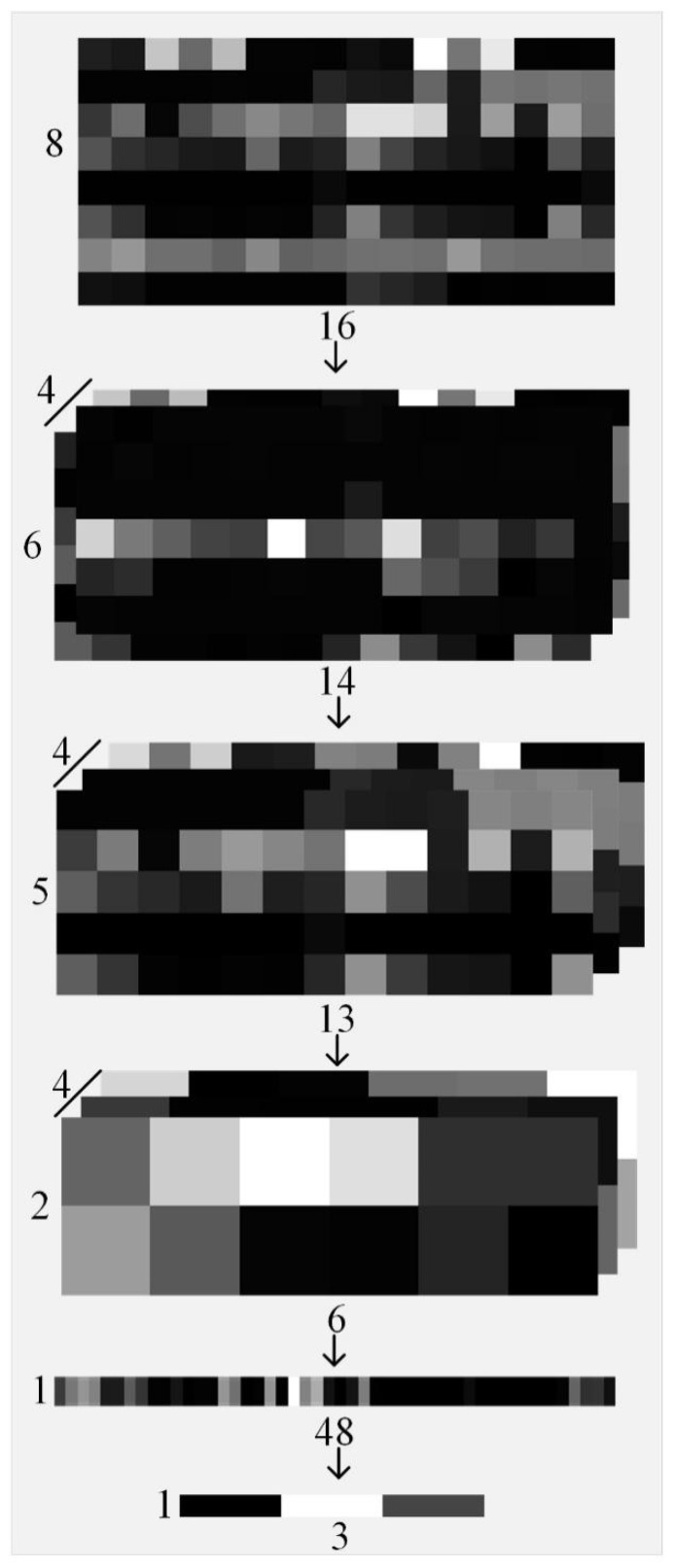
Schematic diagram illustrating the various transformations that occur when a sample multi-feature map is input into the CNN component. An 8 × 16 multi-feature map is input into the CNN component. After being processed by two convolutional layers and a maximum pooling layer, the matrix is flattened into a [1 × 48] vector. This vector then passes through a fully connected layer, which outputs a 1 × 3 vector.

The CNN component of the model, implemented using the PyTorch framework, comprises two convolutional layers, a pooling layer, and a fully connected layer. The first convolutional layer uses four 3 × 3 convolutional kernels with a stride of 1 to execute convolutional operations on the input multi-feature map, followed by the ReLU activation function for processing. The second convolutional layer uses four 2 × 2 convolutional kernels with a stride of 1, which are also processed by the ReLU activation function. The pooling layer utilizes four 2 × 2 pooling kernels with a stride of 2 to execute the maximum pooling operation. After passing through the convolutional and pooling layers, the multi-feature maps are transferred to the fully connected layer. The various transformations that occur as the sample multi-feature map is input into the CNN component are illustrated in Figure 3. The output of the CNN component is further input into the GRU component. The GRU component consists of two hidden layers, each with a dimension of 25, and employs bidirectional GRUs to process the input information. Finally, after passing through the softmax function, the model outputs a probability value that reflects the likelihood of the input sample being a positive sample.

The CNN contains many hyperparameters, which have varying effects on its overall performance, and there are many methods to help adjust these hyperparameters. Based on previous research experience [43], we choose Bayesian optimization to adjust our CNN’s hyperparameters. Specifically, we use the TPE algorithm to optimize the number of convolutional kernels and the learning rate of our model. The number of convolutional kernels is selected from 2^1^, 2^2^, ..., 2^8^ and the learning rate is tuned within a range of 0.0001 to 0.001. Additionally, we utilize the Adam optimizer to adjust the weights and biases of the convolutional kernels. In our model, the batch size is set to 128, the number of epochs is set to 100, and cross-entropy is adopted as the loss function.

#### 2.2.4. Evaluation Criteria

The output of the CNN-GRU model is a probability value, and the closer this value is to 1, the higher the likelihood that the input sample is classified as a positive sample (i.e., an interface residue pair). By sorting the output results in descending order, the top n results represent the n most likely interface residue pairs.

We defined an evaluation indicator, accuracy (${Acc(n)}_{z}$), to evaluate the performance of our model, as shown in Formula (12).

(12) $${Acc\left(n\right)}_{z}=\frac{\sum_{i=1}^{m}{RPT}^{i}\left(n\right)}{m\times TPT}\times 100\%$$

where ${RPT}^{i}$ represents the number of trimer proteins in the test set that is correctly predicted by the model trained on the i-th balanced subset of samples. m is the number of under-sampling instances in the experiment. For a dimer protein, if at least one interface residue pair is accurately predicted within its top n prediction results, the dimer protein is deemed to be correctly predicted. For a trimer protein, if no less than z dimer proteins within it are correctly predicted, then the trimer protein is deemed to be correctly predicted. TPT stands for the total number of trimer proteins in the test set.

We also used four common evaluation indicators (sensitivity, specificity, MCC, AUC) to evaluate the model results. Among them, AUC is an indicator used to quantify the ROC curve, representing the area covered by the ROC curve. The closer its value is to 1, the better the prediction performance of the model. Formulas (13)–(15) provide the methods for the calculation of these evaluation indicators.

(13) $$sensitivity=\frac{TP}{TP+FN}$$

(14) $$specificity=\frac{TN}{TN+FP}$$

(15) $$MCC=\frac{TP\times TN-FP\times FN}{\sqrt{\left(TP+FP\right)\left(TP+FN\right)\left(TN+FP\right)\left(TN+FN\right)}}$$

## 3. Results

### 3.1. Experimental Process

**Figure 4:**
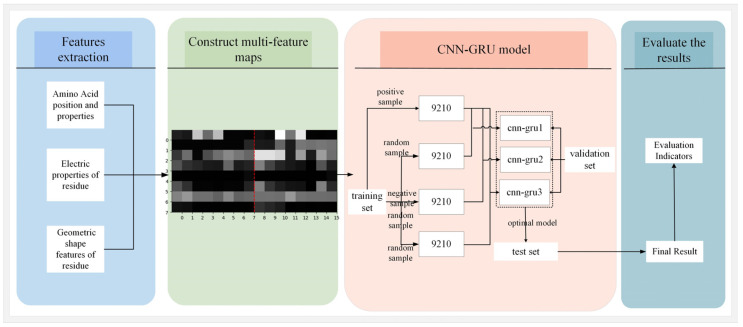
Specific workflow of our experiment.

Based on the data preparation described in Section 2.1, the dataset exhibits a significant imbalance in the number of positive and negative samples. If the dataset is used directly for model training, the prediction results are likely to be biased towards the numerically dominant negative samples, i.e., non-interface residue pairs. However, we are more focused on predicting positive samples, i.e., interface residue pairs. Therefore, we adopt an under-sampling method to construct balanced sample sets, aiming to enhance the predictive performance of our approach [44]. The specific workflow of our experiment is shown in Figure 4.

As shown in Figure 4, the experimental process can be divided into four steps. The first step is feature extraction. According to Section 2.2.1, the physicochemical features, electric features, and geometric features of each amino acid are extracted, with a total of 64 features. The second step is to construct multi-feature maps. That is, for each sample, a multi-feature map is constructed. The detailed steps of this process can be found in Section 2.2.3. The third step is to construct the CNN-GUR model. In this step, we use the under-sampling method to generate three balanced sample sets for model training. The specific process of generating the three balanced sample sets is as follows: there are a total of 9210 positive samples in the training set and we randomly sample three negative sample subsets from the training set, each with the same size as the positive samples; these three negative sample subsets are merged with all positive samples in the training set to generate three balanced sample sets. After training, we obtained three models, named cnn-gru1, cnn-gru2, and cnn-gru3. Then, all samples in the validation set were used to adjust the model’s hyperparameters. Upon the completion of the optimization process, the final hyperparameters chosen were as follows: a learning rate of 0.000814, the number of convolution kernels set to 4, and the number of epochs set to 7. Finally, we input the test set into the optimized models and output the prediction results. The fourth step is to evaluate the results.

### 3.2. Analysis of the Test Set Result

Under the optimal model, we obtained prediction results for the test set and utilized the indicator ${Acc(n)}_{z}$ to evaluate the effectiveness of the model. Here, z represents the number of interaction interfaces in a trimer protein. The stability of trimer protein structures largely depends on the interactions between the residues at the interfaces. If we can accurately identify the correct interface residue pairs for each interface of the trimer protein, this will contribute to the further study of the stability of trimer protein structures. However, the greater the z value, the more difficult it is to predict. Specifically, we assess the prediction results for the test set and summarize the results in Table 1 (where n = 10, 20, and 30).

As shown in Table 1, when each dimer protein provides 10 prediction results and all of the interaction interfaces within a trimer protein need to be accurately predicted, the accuracy of our method is 43%. If the criterion is relaxed to only require the correct prediction of two interaction interfaces within a trimer protein, the accuracy increases to 60%. Furthermore, when the number of prediction results provided by each dimer protein increases to 30, if all of the interaction interfaces within a trimer can be accurately predicted, the accuracy is 75%. In the case in which only two interaction interfaces within a trimer protein need to be correctly predicted, the accuracy reaches as high as 90%.

**Figure 5:**
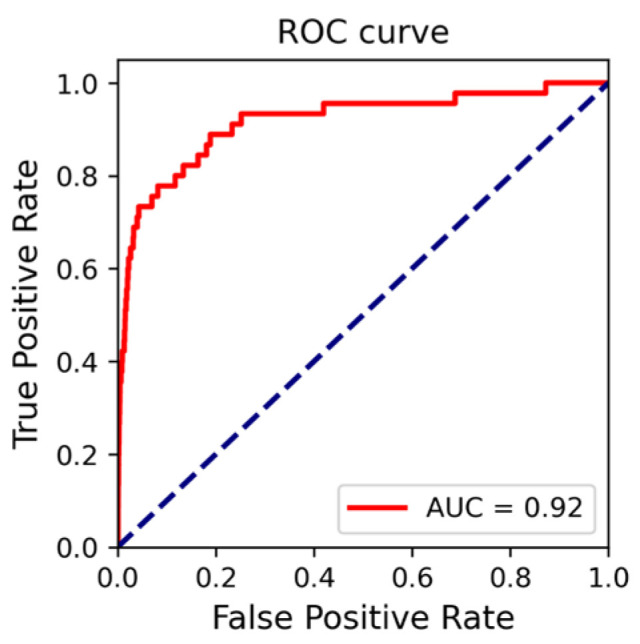
ROC curves and AUC values of the CNN-GRU model on the test set. The red line represents the ROC curve of the CNN-GUR method on the test set. In the legend, AUC stands for the area under the ROC curve.

We used four indicators, the sensitivity, specificity, MCC, and AUC, to evaluate the results of the test set. Our proposed CNN-GRU model exhibited remarkable sensitivity of 0.87 on the test set, indicating a high level of accuracy in predicting positive classes, which is of particular interest to us. Furthermore, the model also achieved specificity of 0.81 on the test set. However, it should be pointed out that, due to the sparsity of positive samples in the test set, the MCC value for the dataset was relatively low, at only 0.09. To intuitively evaluate the performance of the CNN-GRU method, we took the false positive rate as the horizontal coordinate and the true positive rate as the vertical coordinate to draw the receiver operating characteristic (ROC) curve of this method on the test set. As shown in Figure 5, the red line represents the ROC curve of the CNN-GUR method on the test set. The AUC value of this method on the test set is 0.92, further demonstrating its excellent predictive capabilities.

**Figure 6:**
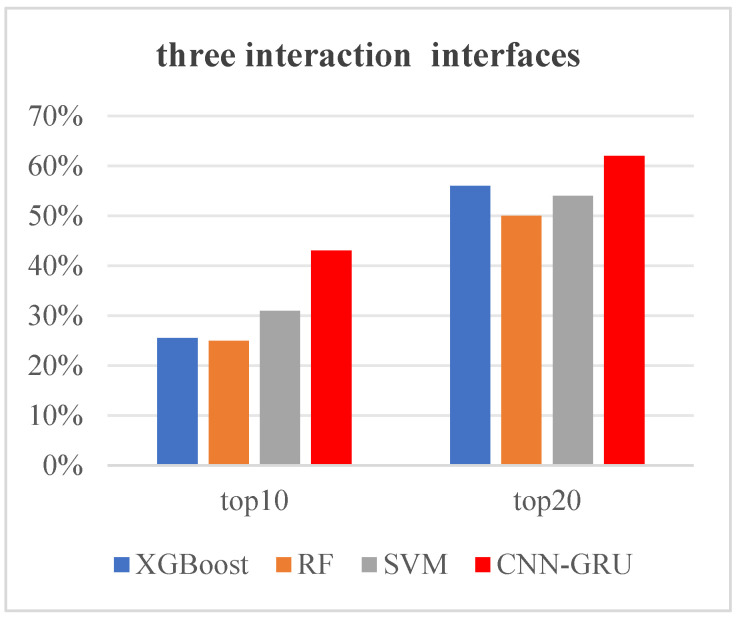
Comparison of the prediction results of the CNN-GRU, XGBoost, RF, and SVM methods on the test set. The horizontal coordinate “top10” (“top20”) indicates that each dimer protein provides 10 (20) prediction results. The vertical coordinate “accuracy” represents the prediction accuracy of the method on the test set when three interaction interfaces within a trimer protein need to be correctly predicted. In the legend, blue, orange, gray, and red represent the prediction results of the XGBboost, RF, SVM, and CNN-GRU methods, respectively.

We also compared the prediction results of our proposed CNN-GRU method with those of the XGBoost, random forest (RF), and SVM methods on the test set, as shown in Figure 6. The four methods used the same training set and test set and maintained consistency in feature usage, utilizing the 128 features extracted in this paper for model construction and prediction. When each dimer protein provides 10 prediction results and three interaction interfaces within a trimer protein need to be accurately predicted, the prediction accuracy is 26% for the XGBoost method, 25% for the RF method, 31% for the SVM method, and 43% for the CNN-GRU method. At this time, the CNN-GRU method is shown to be superior to the other three methods. Furthermore, when each dimer protein provides 20 prediction results and three interaction interfaces within a trimer protein need to be accurately predicted, the prediction accuracy is 56% for the XGBoost method, 50% for the RF method, 54% for the SVM method, and 62% for the CNN-GRU method. Based on the above analysis, it can concluded that the CNN-GRU method performs the best in terms of its prediction performance, while the RF method yields the worst prediction results.

To more comprehensively evaluate the effectiveness of our proposed method, we conducted a comparison with the LSTM method from reference [22] and the two-layer SVM method from reference [23]. The comparison results are shown in Table 2. Specifically, when providing 10 prediction results for each dimer protein and requiring at least one interaction interface within a trimer protein to be accurately predicted, the prediction accuracy is 31.1% for the LSTM method, 76.92% for the two-layer SVM method, and 93% for the CNN-GRU method. Our method is significantly superior to the other two methods. When providing 30 prediction results for each protein and requiring all interaction interfaces within a trimer protein to be accurately predicted, the prediction accuracy is 65.38% for the two-layer SVM method and 75% for the CNN-GRU method. This demonstrates the superior performance of the CNN-GRU method compared to the two-layer SVM method. Additionally, the AUC values for the CNN-GRU method and the LSTM method are 0.92 and 0.65, respectively. In conclusion, the CNN-GRU method exhibits the best overall performance in predicting the trimer protein interface residue pair.

We compared the prediction results of two trimer proteins (1S7O and 3FFD) with relevant experimental references to verify the accuracy of our prediction method. In Ref. [45], it is pointed out that the trimer protein 1S7O consists of two structural domains, with the primary interactions occurring in the second structural domain. The second structural domain contains two helices (residues 75–94 and residues 97–111), within which hydrophobic residues (ILE-85, PHE-86, MET-89, ILE-90, LEU-99, ILE-103, and LEU-106) can form a hydrophobic core with symmetrical ligand molecules. Additionally, residues ARG-82 and ASP-110 can form salt bridges. These structural characteristics contribute to intramolecular and intermolecular interactions. We accurately predicted four interface residue pairs (ILE-105–ILE-109, ILE-105–ILE-105, GLU-101–VAL-81, and ILE-105–ILE-85) within the second structural domain. These four interface residue pairs are all in close proximity to the salt bridge formed by residue ARG-82 and residue ASP-110, so it is inferred that they may play important roles in the formation and stability of the trimer protein 1S7O. In Ref. [46], it is pointed out that, within the C-terminal region of the trimer protein 3FFD, the residue ARG-20 is strictly conserved and forms an interaction with the antibody. Ref. [46] also indicated that the residues GLN-16, ARG-20, SER-52, TYR-59, THR-97, PHE-102, and TYR-104 are binding sites. Gensure et al. conducted photo-affinity cross-linking experiments on residues ranging from 22 to 35 and found that residues PHE-23, LEU-27, and ILE-28 were located near the receptor [47]. We accurately predicted four interface residue pairs (TYR-59–PHE-102, TYR-104–ARG-20, TYR-104–PHE-23 and PHE-102–PHE-23) of the trimer protein 3FFD. Comparing our prediction results with the experimental data from Refs. [46,47], it is demonstrated that the four interface residue pairs that we predicted play important roles in the interactions and stability of the trimer protein 3FFD.

**Figure 7:**
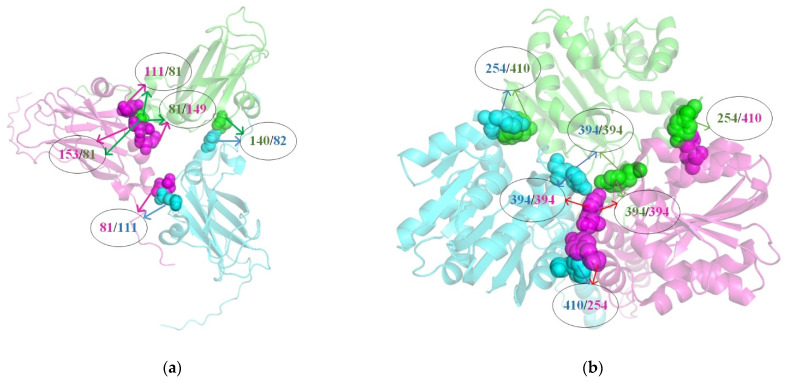
Three-dimensional structures of two trimer proteins, 1AZ7 and 3DLI. (**a**,**b**) display the three-dimensional structures of the protein trimers 1ZA7 and 3DLI, respectively. The different protein monomers are distinguished using varying colors, while the number within the ellipse represents the correctly predicted trimer interface residue pair position.

By analyzing the prediction results and comparing the performance with other computational methods, it is demonstrated that our proposed method is effective in predicting the trimer protein interface residue pair. To visually demonstrate the prediction results, we utilized the PyMOL software (version 3.0.4) to display the three-dimensional structures of the two trimer proteins, 1ZA7 and 3DLI, and clearly annotated the interface residue pairs that we successfully predicted, as shown in Figure 7, in which different protein monomers in the trimer protein are displayed in different colors. Specifically, when providing 10 prediction results for the dimer protein, we successfully predicted five interface residue pairs in the trimer protein 1ZA7 (GLU-140–ARG-82, GLU-81–GLU-111, GLU-81–GLU-153, GLU-81–GLN-149, GLU-111–GLU-81), as shown in Figure 7a. When providing 10 prediction results for the dimer protein, we also correctly predicted six interface residue pairs in the trimer protein 3DLI (PHE-410–TYR-254, TYR-394–TYR-394, TYR-254–PHE-410, TYR-394–TYR-394, TYR-394–TYR-394, PHE-410–TYR-254), as shown in Figure 7b.

**Figure 8:**
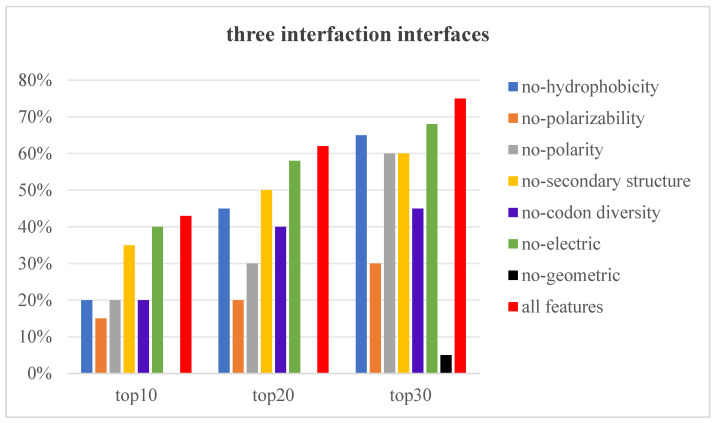
Impacts of different types of features on the prediction results.

The features extracted in this paper can be divided into seven types, namely hydrophobicity features, polarity features, polarizability features, secondary structure features, codon diversity features, electric features, and geometric features. In order to explore the specific impact of each type of feature on the prediction results, we adopted the method of zeroing the value of each type of feature one by one, and we observed the change in the prediction results when the three interaction interfaces needed to be predicted correctly. As shown in Figure 8, when we set the seven types of features to zero one by one, the accuracy showed a decline to varying degrees, which demonstrates that these features have an impact on the prediction results. In particular, when the geometric features are set to zero, the accuracy decreases the most dramatically, so it can be inferred that geometric features occupy the dominant position in these features. When the polarizability features are set to zero, it decreases by two-thirds compared to the accuracy obtained using all features. When the hydrophobicity features, polarity features, and codon diversity features are set to zero one by one, and with 10 prediction results given, the accuracy decreases by half compared to the accuracy obtained using all features.

## 4. Discussion

In the task of studying the interface residue pairs of trimer proteins, our method achieved accuracy of 93% across 10 prediction results when only one interaction interface of a trimer protein needed to be accurately predicted. However, as the prediction complexity increases, the prediction accuracy of our method will also change accordingly. When it is necessary to accurately predict the two interaction interfaces of a trimer protein, the accuracy is 90% when 30 prediction results are provided. When reduced to 20 prediction results, the accuracy drops to 83%, and, when only 10 prediction results are considered, the accuracy further declines to 60%. In addition, when the challenge was extended to accurately predicting three interaction interfaces of a trimer protein, our method’s accuracy was 75% with 30 prediction results, fell to 62% with 20 prediction results, and was only 43% with 10 prediction results. Although this accuracy is improved compared to other computational methods, it is still less than satisfactory. In the future, we can pursue improvements in three aspects in order to potentially further enhance the prediction accuracy. (1) We will introduce more types of features, such as the residue protrusion index, position-specific scoring matrix (PSSM), and so on. (2) We will consider incorporating the flexibility of proteins into the prediction model to more accurately reflect the dynamic behavior of proteins in their actual environments. (3) We will explore deeper structural networks to capture more information about protein interactions. Our model also has limitations. The data used in the model rely on structures resolved via X-ray crystallography, and the under-sampling method used in the model will inevitably introduce biases. The trimer protein interface residue pairs that we accurately predicted may also be key functional sites, which are of great significance in guiding drug development and the design of novel proteins. We also hope to apply our research results to the study of multi-body proteins’ three-dimensional structures.

## 5. Conclusions

In this paper, we utilize amino acid k-interval product factor descriptors to extract the sequence features of amino acids. This method takes into account the influence of other amino acids on the central amino acid based on their distances, combined with their physicochemical properties. Additionally, we also extract the geometric features and electric properties features of residues. Based on the extracted features, we construct an 8 × 16 multi-feature map to represent a sample. Finally, we combine the CNN and GRU to design a CNN-GRU model to predict trimer protein interface residue pairs. When each dimer protein provides 10 prediction results and one protein–protein interaction interface of a trimer protein needs to be accurately predicted, the accuracy of our proposed method is 93%. Our prediction results are consistent with the experimental results, indicating that our method can be used to predict the trimer protein’s interface residue pairs. By comparing our findings with the results of the experimental literature, it is found that the predicted interface residue pairs may be functional sites, which can be further explored in future research. 

## Figures and Tables

**Table 1:** Acc(n)_z_ of test set prediction results.

	n	n = 10	n = 20	n = 30
z				
z = 3		43%	62%	75%
z = 2		60%	83%	90%
z = 1		93%	97%	98%

**Table 2:** Performance of CNN-GRU, LSTM, and two-layer SVM methods based on the Acc(n)_z_ and AUC.

	Indicator	Acc(10)_1_	Acc(30)_3_	AUG
Method				
LSTM		31.1%	—	0.65
Two-Layer SVM		76.92%	65.38%	—
CNN-GRU		93%	75%	0.92

## Data Availability

Data are contained within the article and Supplementary Materials.

## Supplementary Materials

The following supporting information can be downloaded at: https://www.mdpi.com/article/10.3390/nano15030188/s1, Table S1. All residue pairs and interface residue pairs in each protein dimer in the training set. Table S2. All residue pairs and interface residue pairs in each protein trimer in the validation set. Table S3. All residue pairs and interface residue pairs in each protein dimer in the test set. Table S4. Five physicochemical properties for the 20 amino acids.
