# Supplementary material for: Prediction of the Trimer Protein Interface Residue Pair by CNN-GRU Model Based on Multi-Feature Map
Source: Nanomaterials (Basel). 2025 Jan 24;15(3):188. doi: 10.3390/nano15030188 (PMC11821012; doi:10.3390/nano15030188)
Supplement: Supplementary file 1 [file nanomaterials-15-00188-s001.zip › nanomaterials-3422202-supplementary.pdf]

nanomaterials

MDPI

Article

Prediction of the Trimer Protein Interface Residue Pair by CNN-GRU Model Based on Multi-Feature Map

Yanfen Lyu [1,2,3], Ting Xiong [1,4], Shuaibo Shi [2], Dong Wang [5], Xueqing Yang [2], Qihuan Liu [2], Zhengtan Li [2], Zhixin Li [2], Chunxia Wang [6,*] and Ruiai Chen [1,3,4,*]

1 College of Veterinary Medicine, South China Agricultural University, Guangzhou 510642, China; lyuyf@hebeu.edu.cn (Y.L.); bearvet@163.com (T.X.)
2 School of Mathematics and Physics, Hebei University of Engineering, Handan 056038, China; shi258329@163.com (S.S.); yjjmon@163.com (X.Y.); lqh092296@163.com (Q.L.); lzt112496@163.com (Z.L.); lizhixin@hebeu.edu.cn (Z.L.)
3 Key Laboratory of Manufacture Technology of Veterinary Bioproducts, Ministry of Agriculture and Rural Affairs, Zhaoqing Dahuanong Biology Medicine Co., Ltd., Zhaoqing 526238, China
4 Zhaoqing Branch of Guangdong Laboratory of Lingnan Modern Agricultural Science and Technology, Zhaoqing 526238, China
5 School of Mechanical and Equipment Engineering, Hebei University of Engineering, Handan 056038, China; wangdong45@hebeu.edu.cn
6 College of Landscape and Ecological Engineering, Hebei University of Engineering, Handan 056038, China
* Correspondence: wangchunxia@hebeu.edu.cn (C.W.); chensa727@scau.edu.cn (R.C.); Tel.: +86-0310-3968796 (C.W.); +86-020-8755798 (R.C.)

There are 5,493,568 residue pairs in the training set, of which 9210 are interface residue pairs, accounting for about 0.168% of the total residue pairs. The number of residue pairs and interface residue pairs of each protein trimer in the training set is detailed in Table S1. There are 3,123,230 residue pairs in the validation set, including 5411 interface residue pairs, accounting for about 0.1733% of the total residue pairs. The number of residue pairs and interface residue pairs of each protein trimer in the validation set is shown in Table S2. There are 1,725,535 residue pairs in the test set, including 4197 interface residue pairs, accounting for about 0.243% of the total residue pairs. The number of residue pairs and interface residue pairs of each protein trimer in the test set is shown in Table S3. Table S4 describes the values of the five physicochemical properties of the 20 standard amino acids.

Table S1. All residue pairs and interface residue pairs in each protein dimer in the training set.

Protein Dimer	Interface Residue Pairs	All Residue Pairs
1A12_A_B	72	160728
1A12_A_C	67	160734
1A12_B_C	60	160741
1AHS_A_B	70	15805
1AHS_A_C	72	15804
1AHS _B_C	70	15806
1AWI_A_B	4	19039
1AWI_A_P	29	1351
1AWI_B_P	26	1354
1B77_A_B	36	51947
1B77_A_C	37	51947
1B77_B_C	37	51947
1BGX_L_T	118	173761
1BGX_H_T	139	172913
1BGX_H_L	152	43738
1CJD_A_B	151	137116
1CJD_A_C	150	135273
1CJD_B_C	151	136373
1CUN_A_B	31	45337
1CUN_A_C	31	45338
1CUN_B_C	30	45339
1DKG_A_B	157	23700
1DKG_A_D	70	59338
1DKG_B_D	2	56774
1EER_A_B	84	35273
1EER_A_C	55	35303
1EER_B_C	3	45366
1EL6_A_B	182	43081
1EL6_A_C	176	43088
1EL6_B_C	192	43072
1F6F_A_B	80	35604
1F6F_A_C	61	30866
1F6F_B_C	40	32915
1FNS_H_L	160	46919
1FNS_A_L	7	41937
1FNS_A_H	39	43081
1FPO_A_B	16	29224
1FPO_A_C	23	26824
1FPO_B_C	11	26836
1G2X_A_B	31	13892
1G2X_A_C	32	13892
1G2X_B_C	32	13892
1HWG_A_B	109	35586
1HWG_A_C	64	35080
1HWG_B_C	44	37010
1IDP_A_B	77	21531
1IDP_A_C	73	21536
1IDP_B_C	76	21533
1IK9_A_B	172	40192

Table S1. Cont.

Protein Dimer	Interface Residue Pairs	All Residue Pairs
1IK9_A_C	36	5760
1IK9_B_C	30	5430
1J5S_A_B	168	202781
1J5S_A_C	176	202774
1J5S_B_C	172	202328
1JPS_L_T	23	42576
1JPS_H_L	145	45224
1JPS_H_T	62	42538
1JRH_H_L	85	29974
1JRH_H_I	30	17070
1JRH_I_L	29	15836
1KI9_A_B	66	36605
1KI9_A_C	69	36603
1KI9_B_C	68	36796
1KKE_A_B	148	42081
1KKE_A_C	144	42292
1KKE_B_C	164	42066
1L5A_A_B	13	179762
1L5A_A_C	14	179762
1L5A_B_C	6	179770
1LW1_A_B	116	30158
1LW1_A_C	117	29983
1LW1_B_C	114	29642
2ADV_A_B	4	4503
2ADV_A_C	343	79191
2ADV_B_C	202	13630
2AZE_A_B	234	14814
2AZE_A_C	53	6503
2AZE_B_C	53	4391
2B2Y_A_B	100	29474
2B2Y_A_C	28	15022
2B2Y_B_C	21	14513
2B4I_A_B	48	54239
2B4I_A_C	43	53781
2B2I_B_C	48	54240
2BSD_A_B	174	64341
2BSD_A_C	171	64345
2BSD_B_C	171	64345
2CU5_A_B	97	16282
2CU5_A_C	101	16153
2CU5_B_C	94	15908
2DJ6_A_B	60	12014
2DJ6_A_C	60	10965
2DJ6_B_C	62	12013
2E2A_A_B	82	10733
2E2A_A_C	81	10215
2E2A_B_C	84	10212
2FB5_A_B	18	41597
2FB5_A_C	19	41597

Table S1. Cont.

Protein Dimer	Interface Residue Pairs	All Residue Pairs
2FB5_B_C	19	41597
2FM8_A_B	67	16682
2FM8_A_C	107	29373
2FM8_B_C	49	27451
2FVH_A_B	40	9760
2FVH_A_C	47	9754
2FVH_B_C	39	9762
2FZ1_A_B	63	30743
2FZ1_A_C	74	30733
2FZ1_B_C	90	35631
2GDG_A_B	102	12893
2GDG_A_C	102	12894
2GDG_B_C	103	12893
2GMI_A_B	49	20470
2GMI_A_C	15	11537
2GMI_B_C	13	10247

Table S2. All residue pairs and interface residue pairs in each protein trimer in the validation set.

Protein Trimer	Interface Residue Pairs	All Residue Pairs
1STZ	233	297627
2I15	164	42944
2P90	268	216539
2PBQ	184	87018
2R3U	269	112835
2WR5	507	705675
3CC0	170	31400
3EMF	473	37629
3F5C	83	121575
3G65	169	199164
3GI9	231	238055
3M6N	216	211200
3N4G	357	62196
3NAP	593	206244
3O2D	234	125916
3P5J	389	95030
3QKS	320	43559
3R1G	221	210649
3VA2	330	77975

Table S3. All residue pairs and interface residue pairs in each protein dimer in the test set.

Protein Dimer	Interface Residue Pairs	All Residue Pairs
1OSP_H_L	160	46652
1OSP_H_O	30	54718
1OSP_L_O	26	53714
1OY3_B_C	53	15120
1OY3_B_D	38	24640
1OY3_C_D	86	29700
1P32_A_B	93	31122

Table S3. Cont.

Protein Dimer	Interface Residue Pairs	All Residue Pairs
1P32_A_C	95	32032
1p32_B_C	94	30096
1Q5X_A_B	42	25238
1Q5X_A_C	37	24800
1Q5X_B_C	41	24490
1QB3_A_B	7	13447
1QB3_A_C	172	12317
1QB3_B_C	15	12971
1S7O_A_B	12	11130
1S7O_A_C	21	11448
1S7O_B_C	78	11340
1SG2_A_B	44	15369
1SG2_A_C	72	20022
1SG2_B_C	50	15478
1SY6_A_H	37	36792
1SY6_A_L	15	35784
1SY6_H_L	164	46647
1W9Z_A_B	150	66049
1W9Z_A_C	149	65278
1W9Z_A_C	149	65278
1W9Z_B_C	152	65278
1WDJ_A_B	153	28272
1WDJ_A_C	45	34596
1WDJ_B_C	9	28272
1YNB_A_B	197	27889
1YNB_A_C	28	27889
1YNB_B_C	84	27889
1ZA7_A_B	43	24915
1ZA7_A_C	31	24915
1ZA7_B_C	33	27225
2IG8_A_B	98	20306
2IG8_A_C	104	20164
2IG8_B_C	102	20306
2IUM_A_B	86	44521
2IUM_A_C	88	44521
2IUM_B_C	88	44521
2IYO_A_B	90	17176
2IYO_A_C	28	35256
2IYO_B_C	13	11856
2IZW_A_B	88	31862
2IZW_A_C	80	37024
2IZW_B_C	90	37232
2MS2_A_B	39	16641
2MS2_A_C	36	16641
2MS2_B_C	41	16641
3DLI_A_B	86	48841
3DLI_A_C	86	48841
3DLI_B_C	84	48841
3FFD_A_B	150	45570

Table S3. Cont.

Protein Dimer	Interface Residue Pairs	All Residue Pairs
3FFD_A_P	40	3780
3FFD_B_P	26	3906
3OWT_A_B	8	21904
3OWT_A_C	59	2960
3OWT_B_C	31	2960

Table S4. Five physicochemical properties for the 20 amino acids.

Amino Acid	ρ^1	ρ^2	ρ^3	ρ^4	ρ^5	ρ^6	ρ^7
A	0.62	0.046	8.1	−1.302	1.57	0.17	0.50
C	0.29	0.128	5.5	0.465	−1.02	−0.24	−0.02
D	−0.9	0.105	13	0.302	−0.259	1.23	3.64
E	−0.74	0.151	12.3	−1.453	0.113	2.02	3.63
F	1.19	0.29	5.2	−0.59	−0.397	−1.13	−1.71
G	0.48	0	9	1.652	1.045	0.01	1.15
H	−0.4	0.23	10.4	−0.417	−1.474	0.96	2.33
I	1.38	0.186	5.2	−0.547	0.393	−0.31	−1.12
K	−1.5	0.219	11.3	−0.561	−0.277	0.99	2.80
L	1.06	0.186	4.9	−0.987	1.266	−0.56	−1.25
M	0.64	0.221	5.7	−1.524	−1.005	−0.23	−0.67
N	−0.78	0.134	11.6	0.828	−0.169	0.42	0.85
P	0.12	0.131	8	2.081	0.421	0.45	0.14
Q	−0.85	0.18	10.5	−0.179	−0.503	0.58	0.77
R	−2.53	0.291	10.5	−0.055	0.44	0.81	1.81
S	−0.18	0.062	9.2	1.399	0.67	0.13	0.46
T	−0.05	0.108	8	0.326	0.908	0.14	0.25
V	1.08	0.14	5.9	−0.279	1.242	0.07	−0.46
W	0.81	0.409	5.4	0.009	−2.128	−1.85	−2.09
Y	0.26	0.298	6.2	0.83	−0.838	−0.94	−0.71
